# Towards Hybrid Energy-Efficient Power Management in Wireless Sensor Networks

**DOI:** 10.3390/s22010301

**Published:** 2021-12-31

**Authors:** Rym Chéour, Mohamed Wassim Jmal, Sabrine Khriji, Dhouha El Houssaini, Carlo Trigona, Mohamed Abid, Olfa Kanoun

**Affiliations:** 1 Computer and Embedded System Laboratory, National School of Engineers of Sfax, University of Sfax, Sfax 3029, Tunisia; wassim.jmal@ieee.org (M.W.J.); med.abid@enis.tn (M.A.); 2 Measurement and Sensor Technology, Technische Universität Chemnitz, Reichenhainer Straße 70, 09126 Chemnitz, Germany; sabrine.kheriji@etit.tu-chemnitz.de (S.K.); dhouha.el-houssaini@etit.tu-chemnitz.de (D.E.H.); olfa.kanoun@etit.tu-chemnitz.de (O.K.); 3 D.I.E.E.I.—Dipartimento di Ingegneria Elettrica Elettronica e Informatica, University of Catania, Viale Andrea Doria 6, 95125 Catania, Italy; carlo.trigona@diees.unict.it

**Keywords:** wireless sensor networks (WSN), power management, energy saving, microcontrollers, hardware optimization, energy harvesting, scheduling, DPM, DVFS, simulation

## Abstract

Wireless Sensor Networks (WSNs) are prone to highly constrained resources, as a result ensuring the proper functioning of the network is a requirement. Therefore, an effective WSN management system has to be integrated for the network efficiency. Our objective is to model, design, and propose a homogeneous WSN hybrid architecture. This work features a dedicated power utilization optimization strategy specifically for WSNs application. It is entitled Hybrid Energy-Efficient Power manager Scheduling (HEEPS). The pillars of this strategy are based on the one hand on time-out Dynamic Power Management (DPM) Intertask and on the other hand on Dynamic Voltage and Frequency Scaling (DVFS). All tasks are scheduled under Global Earliest Deadline First (GEDF) with new scheduling tests to overcome the Dhall effect. To minimize the energy consumption, the HEEPS predicts, defines and models the behavior adapted to each sensor node, as well as the associated energy management mechanism. HEEPS’s performance evaluation and analysis are performed using the STORM simulator. A comparison to the results obtained with the various state of the art approaches is presented. Results show that the power manager proposed effectively schedules tasks to use dynamically the available energy estimated gain up to 50%.

## 1. Introduction

Wireless Sensor Networks (WSNs) set a very real benchmark for the Internet of Things (IoT) by using the recent technologies to optimize communication and safety systems in hazardous environments [1,2,3]. Thanks to the advantages offered by these technologies, in particular, collecting data in real time with a low cost, WSNs are gaining popularity in several fields of applications such as tele-surgery, intelligent transportation, smart home, industrial control, business sector, virtual reality, law enforcement [4,5,6]. WSNs have to be highly flexible, scalable, interoperable, and reliable. Furthermore, different challenges need to be addressed including improving the energy efficiency, contributing to environmental monitoring and enhancing social services [2]. Indeed, having low power consumption is the primary driver behind the long flow of independent battery-powered devices and to reduce the maintenance cost as most of the nodes are deployed in unattended places.

The cost-effectiveness and energy efficiency could still pose great research challenges in the coming years. Moreover, the exponential complexity of the applications creates a technological gap with the precariousness of the amount of energy available in the sensor nodes. As a result, due to the sensor node’s limited computing capabilities, its load is often confined to trivial calculation. Thus, energy savings can be achieved by using a processing unit with variable-speed processors [7,8,9]. Varying either the frequency or the voltage inevitably leads to a reduction in energy consumption [8]. This is the essence of the DVFS (Dynamic Voltage and Frequency Scaling) technique, which manages the voltage-frequency couple while ensuring a fairly high level of performance. The DPM (Dynamic Power management) technique manages the node’s standby and active states. An efficient management of these power modes contributes to energy resource saving [10].

This paper outlines how the combination of mixed techniques within scheduling algorithms can enrich the current research studies on WSNs and highlight the sometimes ignored insight of these algorithms. However, considering the DVFS and DPM mechanisms for reducing the dissipated power can result in overruns of job deadlines. As a result, the developed Hybrid Energy-Efficient Power manager Scheduling (HEEPS) has integrated a hybrid approach that combines several multi-objective techniques. It relies on task migration techniques, DPM time-out2, where a task is idle for a given time duration switches to the “DeepSleep” state and the DVFS intertask where the speed of each task is fixed and cannot be changed between its different instances. To this end, a global scheduling is deployed through the Earliest Deadline First (EDF) known for its robustness. In addition, we have made some original modifications, which is the combination of Goossens, Funk, and Baruah (GFB) [11] conditions and those of Srinivasan and Belkadi (SB) [12]. In this context, in [13], the authors propose the idea of the HEEPS and its model in the perspective to be used for wireless communications and WSNs. This paper reports the current stage of an activity that has been developed in several years, in particular attention is focused on:

1. A novel WSN energy saving model, called HEEPS, that combines power management and scheduling techniques is developed to schedule real-time tasks. The particularity of HEEPS is its ability to provide a customized energy management algorithm, among time-out 2 DPM and intertask DVFS driven by the GEDF (Generalised Earliest Deadline First) scheduler, to meet the needs of the application. The implemented power model relies on power states as a methodology for power consumption modeling.
2. A comprehensive review of a variety of saving techniques for WSNs is provided. Each technique target to improve the efficiency of a sensor node such as the energy harvesting and clock gating. Moreover, a discussion to demonstrate the efficiency of the proposed algorithm and to compare it with similar works in the literature is carried out.
3. Specification and verification of time constraints through the GEDF scheduler are given combining two conditions of scheduling to avoid the Dhall effect and non-exploited resources:
  (a) Condition of Goossens, Funk, and Baruah
  (b) Condition of Srinivasan and Belkadi
4. Evaluation of the influences of the number of tasks, number of CPU, Worst-Case Execution Time (WCET), Average Execution Time (AET), workload, etc., on saving energy by HEEPS through the STORM simulator. Detailed performance analysis and experimental results are provided showing more energy compared to other existing methods.

The outline of the paper is described as follows. Section 2 includes an in-depth investigation of energy minimisation techniques and a comparison of their performance. Section 3 outlines the energy management model of HEEPS. This Section 4 highlights the configuration used in the simulation experiments. The results and evaluation metrics are discussed in Section 5. A discussion in Section 6 reveals the relevance of our approach to existing work. To conclude the work, a conclusion in Section 7 was given.

## 2. Survey of Energy Optimization Approaches in WSNs

**Figure 1:**
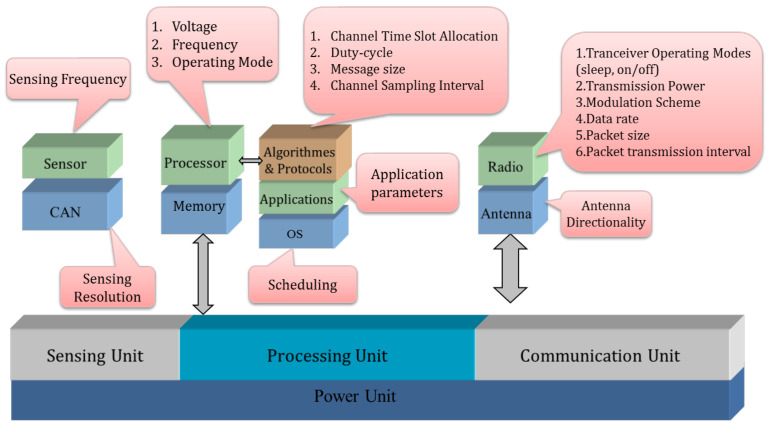
Parameters Involved within a Low-Power Sensor Node.

The high energy dependence weighs on the performance of the WSN, so various techniques have been designed to save the energy. Each unit of the sensor node contributes to the power of the node, as shown in Figure 1. The optimization of energy contributes to the improvement of the performance not only of all the protocol layers of a sensor node, but also involves both the software and the hardware [6].

In the physical layer, the energy can be minimized by reducing the data rate, packet size, and an efficient throughput and through an optimal energy model [14]. In the Medium Access Control (MAC) layer, an energy-efficient duty-cycle design and packet scheduling or an accurate analytical model for the IEEE 802.15.4 CSMA/CA protocol are used to reduce the power consumption [9,15]. Table 1 presents different power optimization strategies and the impact of the considered parameters on the energy minimization [16].

### 2.1. Network Layer

Furthermore, in the network layer, novel routing protocols contribute significantly to reduce the energy [6]. Clustering can bring an important energy saving, whether it is with a periodic variation of the cluster head. It is chosen according to a Round-Robin management policy or the definition of a threshold beyond which the node responsible for sending useful information will switch to balance the node energy consumption [17,18]. Parameters such as residual energy, centrality, and density are to be considered in this case [19,20].

### 2.2. Transport Layer

In the transport layer, the control of congestion and the possibility of its prevention contribute jointly to load-sharing mechanisms to extend the network’s lifetime [21]. Transmission power contributes directly to battery lifetime and network throughput. The greater the range of the nodes, the greater the power required for transmission is. Energy control consists the nodes to ensure minimal energy consumption while maintaining network connectivity. Moreover, data transmission is considered one of the most consuming tasks in WSNs [22]. Sensor nodes are used to send scalar values at regular intervals. This poses a problem of energy conservation, especially if the application is to operate for a long time. Therefore, reducing and optimizing the transmission energy would not make any significant benefit to the data processing. On the other hand, the detection of a change in a vital parameter and the real-time processing of the received information in this case is more important.

### 2.3. Software Level

Energy-saving techniques at the software level include OS-based scheduling, duty cycling, batching, parallel thread processing, and low-power software. Markov Decision Process (MDP) is widely used for resource and power optimization in WSN [23]. It can drains energy with setting a priority scheduling and medium access [24]. The sensing frequency and transmission power are changed with the supply voltage and frequency to recover the node’s new operating state as environmental stimuli change. It can also determine the appropriate time to load a sensor node as in [25].

### 2.4. Processing Layer

The data processing unit consists of a memory, microcontroller, and specific operating system. Due to memory limitations, packets can be deleted before the node can send them to the destination node. Connectivity problems, therefore, arise over time, which can lead to a high latency and subsequently low Quality of Service (QoS). As a result, the power cost of the activation of its processing unit during the operations performed or when reading/writing in memory must be optimized [2].

Dargie [26] studied the relationship between energy consumption, resource utilization, and performance (throughput). The author assumed that the model is more realistic when the usage rates are evenly distributed. The analytical models considered assume that the electrical consumption of a workload can be known in a deterministic way. However, even if a deterministic relationship exists between workload and energy consumption, predicting this workload is tedious. Similarly, the experimental results developed by [27] indicate that there is no significant relationship between the power, CPU utilization, and performance when the server runs with two different workload scenarios, and it is not possible to conclude whether it is because of the DVFS problem or in the workload estimation.

### 2.5. System-Level Techniques

The system level policy covers the hardware level devices where the power can be changed by either varying some parameters or by varying the operating mode. A compromise between energy reduction and performance is achieved by using several operating modes ranging from high performance, high power to low power, low performance. CPU settings include CPU voltage, frequency and power mode.

#### 2.5.1. Undervolting

The undervolting technique was the basis of the work of [28] where the CPU voltage goes below critical thresholds. However, the downside is increased risk of soft and hard errors related to timing violations errors [29]. Due to insufficient supply voltage to drive the processor’s frequency, this risk of error increases with the clock frequency [30]. A further shortcoming is that the same platform gives different results.

#### 2.5.2. Dynamic Voltage Scaling (DVS) Techniques

Authors in [31] describe a cooperative optimization technique that applies DVS and Dynamic Modulation Scaling (DMS) to minimize the power consumption. This technique uses a prediction mechanism to estimate the processor load and the radio communication device based on the log data for a good cooperation. However, it does not rely on the variability of the CPU load parameter which generates temporal irregularity inducing an erroneous result. Therefore, this solution proved to be ineffective to corroborate the performance criteria specific to WSNs.

The paper in [32] aims to minimize the energy of the digital part of a node. To do this, the Quasi Delay Insensitive (QDI) asynchronous logic is used with the software/hardware partitioning of the application. This method implements certain functions of an energy-intensive application on software. The specification of a DVS coprocessor, which is another contribution of this work, allows the control of the processor speed according to a software set point.

#### 2.5.3. Dynamic Voltage and Frequency Scaling (DVFS)

While the frequency (F) is proportional to the voltage (V), the dynamic power is proportional to the square of the voltage. Thus, lowering both (F, V) induces a cubical drop in power [8]. This DVFS hardware strategy hinders the overall performance and brings overheads penalties due to the changes of the (F, V). Software solutions come into play to counter this limitation, including task migration, injection of idle cycles and scheduling that balances the load between the processors to enhance overall energy consumption [33].

The EA-DVFS algorithm of [34] delays task execution if sufficient energy is not available. The system runs at high speed if not. However, the energy value is counted on a per-task basis. Until the system’s remaining running time at maximum speed exceeds the task’s relative deadline, the system assumes sufficient energy. If the system has an energy of around 1% it will operate at a maximum speed, which will deplete the energy more quickly. When the voltages are selected and the tasks are scheduled, the EA-DVFS algorithm considers only one task in the queue of ready jobs instead of considering them all. As a result, slack time or periods of inactivity are not fully exploited to save energy. It should be noted that our contribution covers all the tasks ready to elect the one that will be executed and is based on several couples of voltage/frequency and not only the maximum and minimum value.

In [35], DVFS is implemented in MSP430, but the disadvantages of this strategy are the synchronization mechanisms that should be considered with caution.

#### 2.5.4. Dynamic Power Management (DPM)

Energy efficiency can be maximized by applying techniques. As energy is available, the system must also be able to adapt its consumption, if necessary by shifting the most energy-intensive tasks to more convenient periods, or even by switching the system to a less energy-intensive state when they are idle or at standby or partially used, while limiting the impact on performance [36].

DPM to reduce the static voltage and DVFS to reduce the dynamic power have been applied together in [35,37,38]. Indeed, non-compliance with time constraints can affect the efficiency of the network. This solution would solve only partially the problem and would generate huge costs.

#### 2.5.5. Off-Line Techniques

For the reason of simplicity, many researchers used off-line techniques [39]. This optimization technique is based on MIxed Linear Programming (MILP) and a Directed Acyclic graph (DAG). It militates for a combination of DVFS and DPM for a set of dependent tasks (precedence constraints), periodic, with a deadline on request and running on multiprocessor systems. Because of the extreme disparity in application scenarios that are constantly and dynamically changing, this solution is inappropriate and sub-optimal facing the importance of energy efficiency. In addition, it is not able to grasp the dynamic behavior of the entire software/hardware system and incurs the tasks with a worse case behavior (WCET), which is not efficient, leading to a negative energy balance especially when the dynamics of the system are important.

### 2.6. Energy Harvesting Solutions

The emerging trend whereby demand for energy is satisfied, is deploying energy harvesting solutions [40,41]. The motes provide extra energy by gathering, kinetic (wind, waves, gravity, vibration), piezo, electromagnetic (radio frequencies, photovoltaic), or thermal energy (solar, temperature gradients) for an unlimited amount of time. Harvested energy from irregular and fluctuating sources such as wind power, photovoltaics, capacitive methods, etc., are low and not continuously available to meet the demand of a power system [41,42,43,44]. Indeed, they are often produced away from the consumer places, where local infrastructure is less robust. In smart health applications, recovering energy brings risks and skin infections [43]. Consequently, it would be wise not to equip the network more but to implement an efficient energy management strategy allowing reliable control of the nodes.

The contributions of [42] focus on an energy manager based on a multiple converter of energy sources. The energy storage system consists of two capacitors to provide a rapid initialization capability. To solve this issue, many researchers have proposed various methods that are classified according to two antagonistic approaches. First, some studies proposed adapting the performance to meet the requirements of the lifetime or the availability of energy. Although promising, these efforts have been examined rather roughly through a high level abstraction, by adapting the duty cycle or the sampling frequency [45,46], except that they do not provide an independent energy-based configuration. Second, high-level power management mechanisms drive the hardware directly without any isolation, resulting in a time-consuming and hard to debug configuration. Additionally, this solution suffers from high losses due to variable workloads.

The strong impact of weather conditions, such as changing from night to day or vice versa, limits the performance of the energy harvesting. Typically, the relay node maintains a high QoS throughout the day, but extremely low QoS at night. The problems associated to those techniques range from erroneous hypotheses to unforeseen difficulties and unpredictable environmental dynamics. In conclusion, the non-stability of these systems dependent on climate and on the environment. The importance of wake-up time interval from $T_{EI}$ to $T_{NEI}$ and the degradation of performance and QoS affected negatively the expansion of this type of system.

### 2.7. Comparison of Energy Saving Techniques

Many research works have tackled the matter of energy minimization [4]. Some of them have focused on the DPM technique [36,47,48,49,50,51,52,53,54]. The implementation of the DVFS in WSNs is widely applied [33,55,56,57]. Only few papers combined DPM with DVFS in WSNs such as in [35,37]. Additionally, the scheduling has been considered in [49,58]. Table 2 describes the main techniques used to optimize the energy consumption in the WSN.

The outcome of this comparison is both DPM and DVFS have a great impact on power saving while respecting the scheduling constraints mainly for real-time multiprocessor systems [62]. In this paper, the focus is on using strategies that vary during the execution and are adapted to any type of application whose level of consumption is likely to increase. To this end, we have chosen an on-line energy management techniques that best optimize energy consumption and maximize QoS.

To meet the performance constraints and workload variations, DVFS tends to be combined with scheduling [33,57]. Therefore, slowing down the speed of execution is more optimized in energy terms if the deadlines of the tasks are respected. If there is a slack in the execution time, executing as slow as possible, while just meeting the timing constraints is more dynamic-power-efficient than executing as fast as possible and then idling for the remaining time. However, the delay of a circuit also depends on the supply voltage. Thereby, by reducing the supply voltage, although we can achieve a cubic power reduction, the execution time increases. The main challenge in achieving the power reduction through voltage and frequency scaling is to overcome all timing constraints.

**Figure 2:**
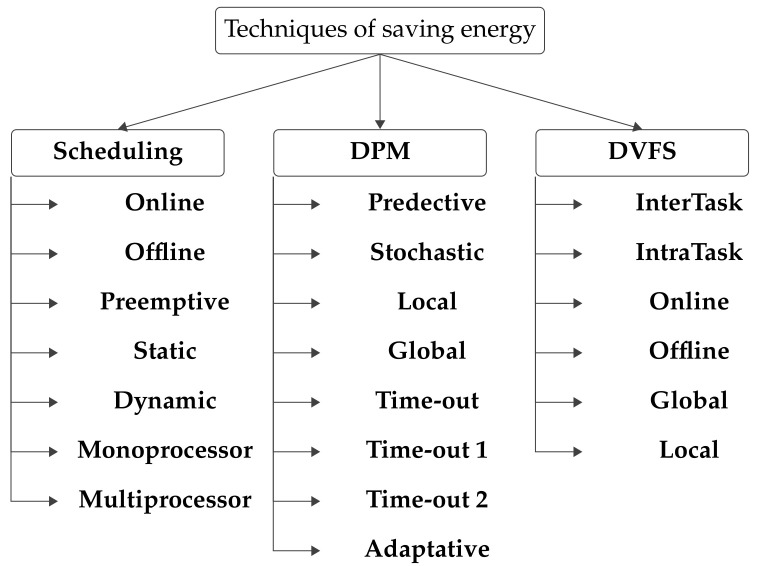
Power Management Taxonomy at CPU Level.

In Figure 2, a power management taxonomy for CPU-level used in HEEPS like DPM, DVFS and scheduling is established. After presenting a palette of the main low-power techniques of embedded systems, we will present the power consumption reduction model adopted for the sensor nodes.

## 3. HEEPS: A Hybrid Energy-Efficient Power Manager Scheduling

Applying several energy management strategies at once provides more energy benefits than settling on one method. Therefore, we propose an online HEEPS power manager that incorporates three energy management strategies. This results from the trade-off between time constraints and power-modes.

**Figure 3:**
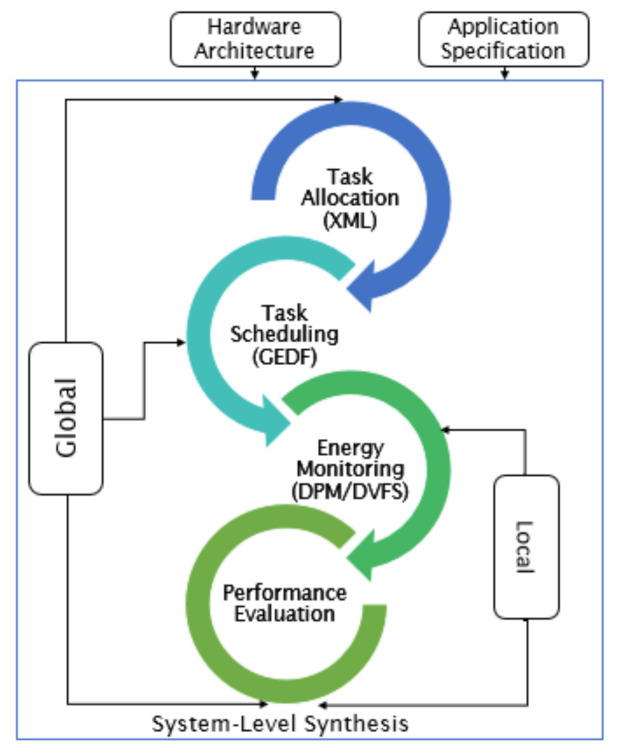
HEEPS Phases Integration.

By providing a functional modeling of a low power energy manager, the need for manual intervention is minimized. The advent of functional and structural failure risks is eliminated through the use of a robust verification methodology. A low consumption system level model is presented that acts on the wireless sensor network both at the local node level and at the global level, i.e., at the network level. The development process is described in Figure 3. The “Task Allocation” phase distributes tasks to all nodes. Once the inputs (time settings, tasks number, or nodes number) are set, the GEDF scheduler sorts the tasks in the “Task Scheduling” phase. However, selecting the application-specific energy management technique is done in the “Energy Monitoring” phase at the local level. The “Performance evaluation” phase involves measuring the performance metrics.

**Figure 4:**
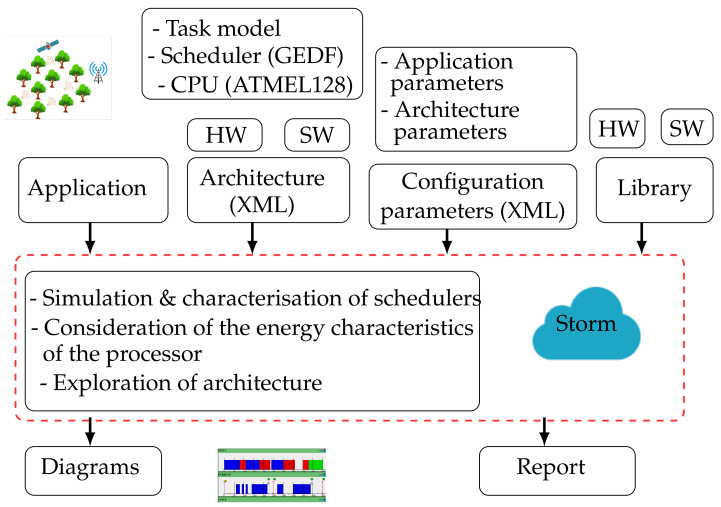
Backend Design of HEEPS Model.

This model is built on low-power typical processor characteristics such as frequency, voltage, and timing constraints. The backend design of HEEPS model is illustrated in Figure 4. Through the hardware (resources) and software architecture (tasks) and the scheduler, the STORM simulator can allocate and schedule the tasks according to GEDF [16]. Overheads and delays are supported by the scheduler as well. The XML file is loaded with the inputs before the simulation. The results are displayed as Gantt charts or as PDF reports to debug the model and show the behavior of the network. HEEPS is used as an interlayer in-between the application level and the hardware resources for energy manager portability.

**Figure 5:**
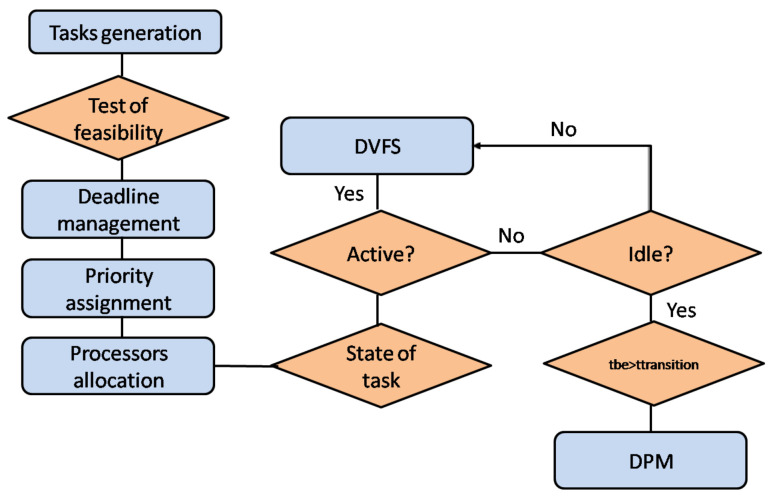
Flowchart of Execution of HEEPS.

The on-line characterization allows to predict the mode of consumption and the appropriate voltage/frequency couple at a given moment. The increase in the execution time when tasks are active, through the DVFS, contributes in particular to the minimization of the period of inactivity, allowing more efficient use of low consumption states. DPM is applied during idle intervals while DVFS is applied when the task is running as in Figure 5.

Algorithm 1 gives a formal description of both DPM and DVFS strategies with the simulator STORM when the scheduling constraints are satisfied.
**Algorithm 1:** HEEPS Implementation
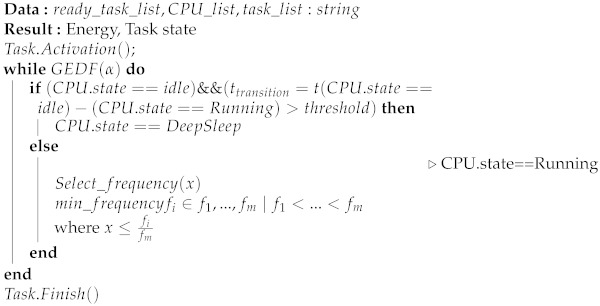


The choice of the Actual execution Time (AET) in STORM is made according to the Real Execution Time (AETRule). It can be equal to WECT or random comprised between the BCET, and the WCET selected after each new activation. WCET means “Worst Case Execution Time” and BCET stands for “Best Case Execution Time”.

## 4. Simulation Setup

To extend the node lifetime, new utilities are needed to facilitate the development of sensor networks. Pre-prototyping tools, for example, must provide fast and reliable results at a low cost to guide the design or the validation choices of the appropriate hardware. The simulator must not only imitate the real world, but also take into account certain metrics that certainly have significant impacts on network characteristics. These metrics include the time (latency, lifetime, idle time, transmit/receive time, etc.), throughput, power consumption, and quality of service (network failure, coverage, and packet loss). The simulation models the node’s behavior since they use profiling rules and describe the node as a finite state machine. The feedback received contributes to the energy efficiency upgrade.

The study of more than 25 simulation environments in [16] conducted to the STORM simulator. Its multiple advantages correspond to tracking the realistic temporal evolution of tasks where each CPU is equivalent to a real node. It also allows a large-scale simulation with several nodes.

Although the transitions add extra costs and risk tasks missing their execution, they are rarely invested in the research work. Additionally, each power-mode refers to a particular energy and delay, overlooking them brings error, hence, we need a precise modeling and not approximation. For this reason, the multitasking system used promotes the use of scheduling, otherwise it lengthens the ET in case of DVFS. Predetermination of system behavior is an onerous task, which is why we chose the online scheduling.

**Figure 6:**
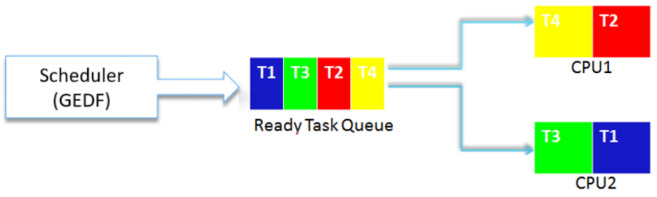
Tasks Scheduling Scheme of HEEPS.

This approach is also important given the evolving nature of energy. Although our simulations are restricted to a periodic model, they can be extended to an aperiodic or sporadic model. The GEDF scheduler is based on a periodic, preemptive task set where the deadline is equal to the period with no task affinity (tasks can change CPU). The GEDF performs both the allocation of tasks to processors and makes scheduling decisions. After each period $T_{i}$, the GEDF scheduler must keep track of the deadlines values $D_{i}$ and check if any new task with a smaller deadline value arrives as shown in Figure 6. In a context of global scheduling, and since some task sets may be not schedulable even though they have low utilization (much less than the number of processors) and in order to respect temporal constraints, our scheduler is distinguished by an association of conditions of Goossens, Funk, and Baruah and Srinivasan and Belkadi. Indeed, comparing the total usage rate $u_{Total}$ to the CPUs number leads to a set of non-schedulable tasks (starvation).

Therefore, other conditions should be applied to test the schedulability of the GEDF, including the threshold on $U_{max}$ which are those of Goossens, Funk, and Baruah (GFB) [11] and Srinivasan and Belkadi [12]:

- In the GFB rule, the total utilization ratio threshold is:
  (1) $$u_{max}\leq m\cdot \left(1-u_{max}\right)+u_{max}$$
  where $u_{max}$ is the maximum utilization rate of task and *m* is the number of CPU (in our case m=5). We found 1.406, which is well below the GFB threshold, which is 3.8.
- The utilization rate of Srinivasan and Belkadi for each task is
  (2) $$u_{max}\leq \frac{m}{2\cdot m-1}$$
  Tasks are scheduled on *m* processors as long as the total utilization is:
  (3) $$U_{total}\leq \frac{m^{2}}{2\cdot m-1}$$
  As a result, we have $U_{i}=0.3<0.55$ and $U_{total}=1.406<2.77$.

Table 3 presents the temporal characteristics of tasks. A set of 10 periodic tasks, where $T_{i}$ represents the task *i* and $T_{i}=D_{i}$. The scheduling test is verified under the GEDF scheduler. We relied on the system’s energy levels and node usage profiles to decide which policy to use between DPM and DVFS techniques.

A description of the the simulation metrics is given in Table 4.

## 5. Performance Evaluation of HEEPS

To supervise the functionalities of HEEPS, various simulations and measurements are performed. Several parameters are taken into account to measure the power consumption of the CPU.

**Figure 7:**
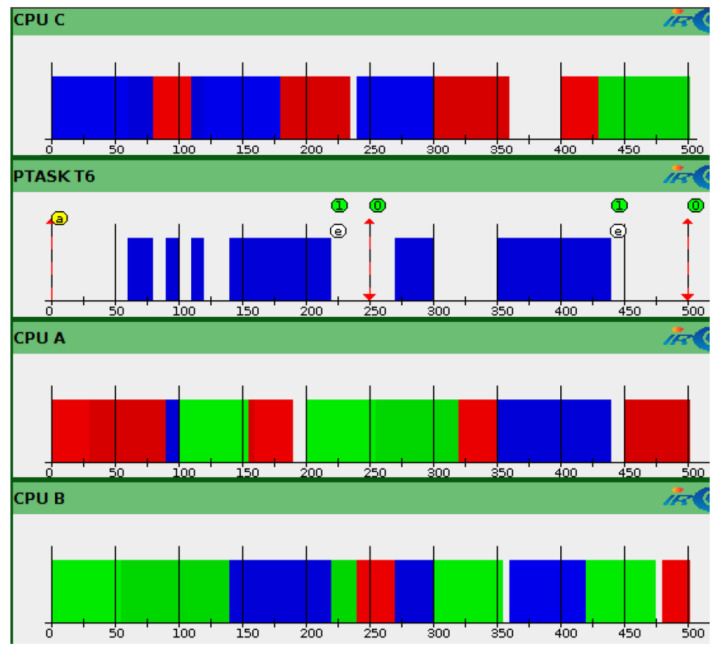
Gantt Diagrams of Simulation Results.

Figure 7 shows the Gantt diagrams of the execution of the different tasks. To validate the results of HEEPS and the GEDF scheduler, we check the time parameters through the STORM simulator. Time is displayed in units of 50 ms (*X*-axis). Each task is represented by a different color. They are activated simultaneously at t=0. Tasks are migrated between the different queues of the ready tasks in order to have always *m* CPU and the *m* earliest task deadlines ready to be executed on these *m* processors.

Various frequencies and voltages are listed in Table 5. The variation of the frequency brings a gain of energy especially when the standby mode is not allowed. Running the processor at a frequency equal to 4 MHz, this results in a significant reduction in the energy consumption compared to $f_{max}$ close to 52%.

**Figure 8:**
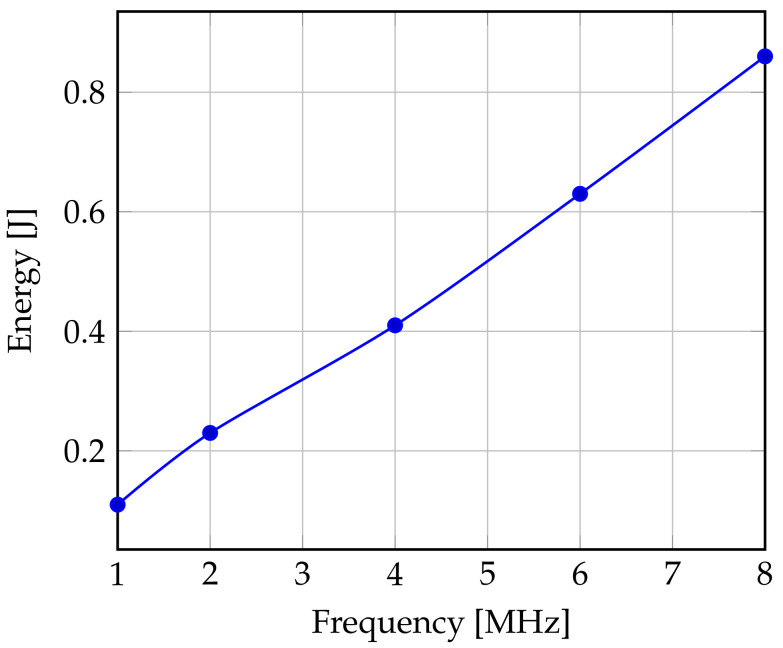
Effect of Reducing Frequency on Consumption.

Figure 8 shows that if the CPU frequency changes with DVFS from $f_{max}=8$ MHz to 6 MHz, the energy is 0.86 J (5.5 V) and 0.63 J (4.05 V), respectively. The energy gain is then 26%. For this purpose, the lower the frequency, the greater the gain, when the ’ET’ is increased, the idle time is reduced, which can lead to a delay in execution justified by numerous instructions against a limited CPU processing capacity. The decrease of the frequency induces a nonlinear minimization of the energy dissipation but it achieves an energy saving, as shown in the Figure 8, between 26% and 52%.

**Figure 9:**
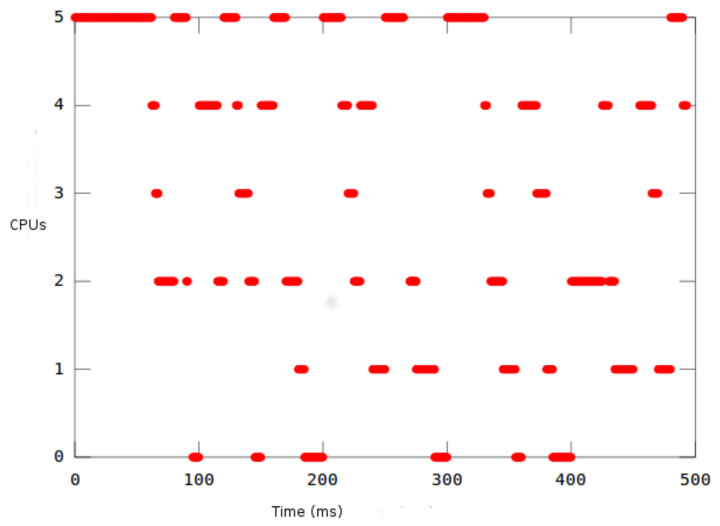
Evolution of the Number of Active Processors.

Figure 9 describes how the number of active processors evolves during the simulation period (500 ms). This number varies between a minimum equal to 0 and a maximum, which is the number of theoretical processors satisfying the feasibility test of GEDF (equal to 5 in our case). It should be noted that this variation follows the workload in the system. By adding more processors, the CPU time and last scheduled task timeout are both shortened. The more we adjust the frequencies, the more the energy gain is important according to the simulation, globally.

The variation in the number of tasks has a considerable impact on energy consumption. To do this, we started several simulations by modifying the number of tasks. The primary factor in energy consumption variations is the actual workload. As more tasks are performed, more dynamic latencies are generated and therefore more opportunities for DVFS to regain energy. HEEPS may even result in energy savings more than any stand-alone policy in some cases. Every time the tasks number rises, the power consumption rises since the processors in this case are more solicited. To evaluate the reliability of the process, many tasks are deployed first with an AET equal to the WCET and then with a random value.

**Figure 10:**
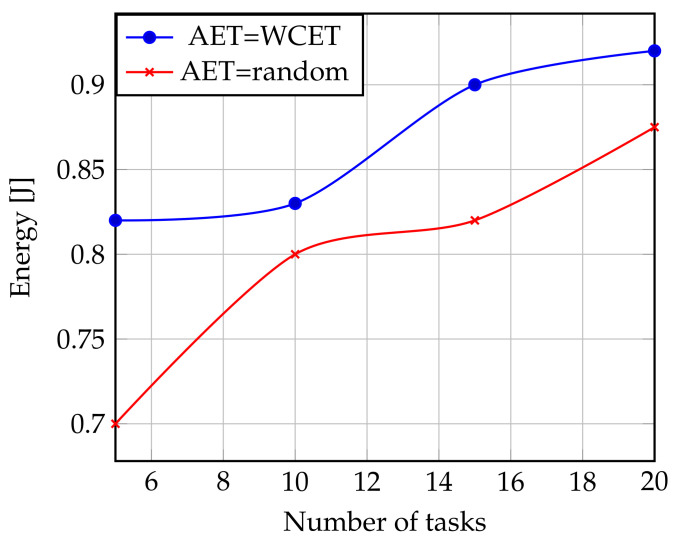
Influence of the AET and Task Number on Energy.

Figure 10 states a gain of the order of 11.82% with the same assumptions against 19.5% gain when the value of the AET is random. The use of the WCET value guarantees us an energy gain, but it is obtained at the expense of performance.

## 6. Discussion

Interacting at the node level and further upstream at the network level significantly reduces energy. This section assumes the role of highlighting the added value of the developed HEEPS energy manager. Increasing the reliability of the electrical supply contributes to the good functioning of the sensor nodes and to a better quality of service, and stops the network breaking. This fully supports our strategy. Indeed, a one-off power reduction strategy is insufficient to affect significantly a CPU’s power consumption profile (often because of the transition costs). HEEPS crosses over DPM, DVFS, and GEDF to achieve better energy savings.

The opportunities offered by HEEPS are numerous. It is able to adapt to all types of processors of sensor nodes and to give results closer to reality. It provides a portable solution on several platforms and simulators by changing some components (CPU frequencies, penalties of transition, power-modes, etc.). After identifying, reviewing, and validating a specific scenario, the solution can be used to explore other instances that fall within the same logic without complicated parameterization. Tracking the node’s behavior would be a way to create a relevant energetic and performance-assured strategy. Therefore, HEEPS is a highly modular system providing utmost scalability and flexibility.

Although most power manager are intended for energy harvesting like [42,63], they remain weak and the trend scenario is not viable despite the advances recorded on energy-gathering components. A disadvantage of Kan-PM proposed by Kansal et al. [63], is its low response to the change in energy (unable to take into account the reloading of the storage unit), which induces an error when measuring the available energy.

DVFS alone is not the best strategy to apply to modern processors, but it could be used with other energy management techniques. The DPM algorithm may malfunction if $T_{transition}$ it is usually longer than the idle time available. Therefore, in our mixed approach, we have taken these two considerations into account. The techniques of [34,37] target to prolong the execution time as much as possible. As illustrated in [37], structuring energy management techniques exclusively on two frequencies of voltage/frequency leads to difficult design errors. They cause an imbalance in the energy manager’s decisions and the real execution states. This problem was overcome with HEEPS by taking into account discrete frequencies and voltage.

Table 6 summarizes, for each solution, the corresponding features and metrics. The work of [39] is based on precedence constraints. It results usually in a cumulative delay in execution resulting in deadlines violation. That is why we have privileged tasks with deadlines on request. Although [26] is limited to three power modes, there are five low-power states in our framework. We assume a deep sleep state with a $T_{transition}$ lower than or equal to the idle interval length.

The comparison of our work with the other proposed solutions allowed us to consolidate our contribution and take into account its limits also in our future work. The energy model is implemented in STORM, encapsulating the power consumption of all operating modes of the CPU component and the transitions between those different power modes. In addition, in order to take advantage of HEEPS, we have developed several simulations based in particular on the variation in the number of tasks, the utilization rate and the best and worst execution time ratio to explore more the reliability of the adopted algorithms. Results show that the power manager proposed effectively schedules tasks to utilize the dynamically available energy estimated gain up to 50%.

## 7. Conclusions

HEEPS acts on the local and global level when most of the works focuses on the network aspect of the WSNs. Additionally, more power modes are considered compared to other researches. Similarly, the power manager stands out by its hybrid aspect and its combination of several methodologies DPM (time-out2) DVFS (intertask) and GEDF scheduler contributing to more optimality in terms of scheduling. Two conditions of scheduling are combined, which are Goossens, Funk, and Baruah and Srinivasan and Belkadi and non-exploited mainly under the WSNs context. Besides, the STORM simulator is selected to focus more on its many advantages. Furthermore, the STORM features are extended by improving its performances and functionalities. Finally, in the discussion part, HEEPS is compared with recent energy-aware works to highlight the originality of our work. The simulation validates the effectiveness of the results. HEEPS minimizes the total energy consumption ranging from 50% to 80%.

## Figures and Tables

**Table 1:** Impact of energy saving technologies in WSN.

	Technique	Performance	Overhead	Architecture	Design	Validation	Power Type
Power Mode Based Strategies	DPM	++	+−	++	++	++	Static & Leackage
	Clock Gating	+−	−	−	−	−	Dynamic
	Power Gating	++	+−	++	++	++	Leackage & Standby
Multi Voltage Design Strategies	MVS ${}^{1}$	++	+−	++	+−	−	Dynamic
	SVS ${}^{2}$	+−	−	−	−	None	Dynamic
	DVFS	++	+−	++	++	++	Dynamic

− Low, +− Medium, ++ High; ^1^ Multi-level Voltage Scaling; ^2^ Static Voltage Scaling.

**Table 2:** Comparison of Energy Saving Techniques.

	[51,52,53,54]	[27,35,55]	[26,49]	[59]	[28,29,30]	[35,37,38]	[58]	[23,24,25]	[60]	[61]	[33,57]	HEEPS
DPM	x		x	x		x						x
DVFS		x				x					x	x
Undervolting					x							
Scheduling			x				x			x	x	x
MDP				x				x				
Clock Gating									x	x		
Power Gating									x			

**Table 3:** Example of System Timing Requirements (ms), where n=10 tasks m=5 (CPU).

	T1	T2	T3	T4	T5	T6	T7	T8	T9	T10
Period	80	100	120	150	200	250	80	80	80	80
WCET	10	30	20	15	20	5	10	15	12	7
BCET	1	3	4	3	4	6	1	3	4	3
Deadline	80	100	120	150	200	250	80	80	80	80

**Table 4:** Simulation Evaluation Metrics of HEEPS.

Metrics	Values
Time frame	1000 ms
Precision	$10^{-9}$ s
Number of tasks (*n*)	2, 5, 10, 20
Number of Processors (*m*)	2, 3, 5, 10
$\frac{U_{total}}{m}$	70, 75, 80, 85, 90, 95, 97.5, 100
Execution time	WCET and AET
Failure to meet deadlines	Task abortion
Scheduler	GEDF
Distribution of periods	[2, 100] ms
Penalties Overheads	Applied to DPM and not for DVFS

**Table 5:** Frequency/Voltage Couples Supported by ATmega128L.

F (MHz)	8	6	4	2	1
V (V)	5.5	4.05	3.6	3.15	2.7
DVFS_factor	1	0.75	0.5	0.25	0.125
Energy (J)	0.86	0.63	0.41	0.23	0.11

**Table 6:** Comparison of HEEPS with Existing Techniques.

References	Algorithms	Tasks	On-Line	Off-Line	Energy Harvesting	Scheduler	Penalty of Transition	Migration	Overhead
[26]	DPM	Periodic		x		FIFO	No	Non	No
[49]	DPM	Periodic	x		x	EDF	x	No	x
[35]	DVFS		x		x	-	-		No
[27]	DVFS	Periodic							No
[34]	EA-DVFS	Periodic, Preemptive	x		x	EDF	-	No	Negligible
[57]	DVFS-HESS ${}^{1}$	Uniform, synthetic	x		x	LSA	x	-	No
[37]	DPM-DVFS	Periodic, Preemptive	x					Non	Non
[39]	DPM-DVFS	Periodic, dependent		x	-	Time-Triggered		x	x
[42]	BQS-PM ${}^{2}$	Periodic	x		x	-	x	No	Yes
[63]	KAN-PM	Periodic		x	x		-	No	
[64]	iMASKO	Sporadique		x	x		x	-	-
HEEPS	DPM-DVFS	Periodic, Preemptive, Independent	x		-	GEDF	x	Yes	No

^1^ Hybrid energy storage system; ^2^ Quality of service-based Power Manager.

## Data Availability

The data presented in this study are available on request from the corresponding author.
